# Revealing viral hepatitis epidemiology in the Democratic Republic of Congo: insights from yellow fever surveillance reanalysis

**DOI:** 10.1186/s41182-025-00687-8

**Published:** 2025-02-05

**Authors:** Patrick Mukadi-Kakoni, Yannick Munyeku-Bazitama, Gracia Kashitu-Mujinga, Marguerite Manwana-Pemba, Niclette Zenga-Bibi, Patient Okitale-Talunda, Christelle Mbelu-Kabongo, Fleurette Domai-Mbuyakala, Elisabeth Pukuta-Simbu, Pierre Mutantu-Nsele, Yoshinao Kubo, Sheila Makiala-Mandanda, Steve Ahuka-Mundeke, Koya Ariyoshi, Jean-Jacques Muyembe-Tamfum

**Affiliations:** 1 Department of Clinical Medicine, Institute of Tropical Medicine, Nagasaki University, Nagasaki, Japan; 2 Institut National de Recherche Biomédicale, Kinshasa, Democratic Republic of the Congo; 3 Département de Biologie Médicale, Faculté de Médecine, Université de Kinshasa, Kinshasa, Democratic Republic of the Congo; 4 Program for Nurturing Global Leaders in Tropical and Emerging Communicable Diseases, Graduate School of Biomedical Sciences, Nagasaki University, Nagasaki, Japan; 5 Division of Global Epidemiology, International Institute for Zoonosis Control, Hokkaido University, Sapporo, Japan; 6 Faculty of Medicine, University of Kikwit, Kikwit, Democratic Republic of the Congo

**Keywords:** Hepatitis, Yellow fever surveillance, Seroepidemiology, Acute febrile jaundice, Democratic Republic of Congo, Public health, Viral hepatitis

## Abstract

**Background:**

Yellow fever surveillance systems are designed to identify cases of acute febrile jaundice, a clinical syndrome used to monitor the emergence of yellow fever outbreaks. However, this syndrome has diverse etiologies, particularly viral hepatitis. This study investigates the seroepidemiology of viral hepatitis A (HAV), B (HBV), C (HCV), and E (HEV) among cases initially suspected to be yellow fever, aiming to elucidate the epidemiology of viral hepatitis in the Democratic Republic of Congo (DRC) and provide insights for improving public health interventions.

**Methods:**

A retrospective cross-sectional study was conducted using serum samples collected between 2017 and 2018 through national yellow fever surveillance in the DRC. Samples from individuals testing negative for yellow fever were tested for IgM antibodies against HAV, HBc, HCV, and HEV and HBs antigen using validated ELISA kits. Acute HBV infection was defined by both HBc IgM and HBs antigen positivity. Multivariable logistic regression was used to assess the association of demographic, geographic, and environmental factors with each hepatitis type.

**Results:**

Among 1239 participants (58.8% male; median age: 16 years), seroprevalence was 16.1, 11.2, 5.0, and 3.1% for HAV, HBV, HCV and HEV, respectively. HAV prevalence was highest in the youngest age group and rural residents. In contrast, the youngest group was most protected from HBV. HCV prevalence was highest in the oldest age groups. HEV exhibited higher prevalence during the dry season and in a humid subtropical climate. Several provinces were identified as hotspots of HAV, HCV and HEV.

**Conclusions:**

Viral hepatitis is a major cause of acute febrile jaundice in the DRC with notable geographic and seasonal trends. National yellow fever surveillance is a valuable resource for understanding hepatitis epidemiology, though careful interpretation is necessary. Tailored interventions are required for mitigating the burden of viral hepatitis in each province.

**Supplementary Information:**

The online version contains supplementary material available at 10.1186/s41182-025-00687-8.

## Background

Yellow fever, a viral hemorrhagic fever caused by the yellow fever virus and transmitted by *Aedes* mosquitoes, remains a significant global health concern, particularly in sub-Saharan Africa and South and Central America, despite the availability of a highly effective vaccine [1]. The World Health Organization (WHO) recommends a syndromic approach for surveillance in endemic regions, diagnosing acute febrile jaundice as yellow fever based on clinical criteria, though laboratory confirmation remains essential for accurate diagnosis and effective disease management [2–4]. However, acute febrile jaundice, characterized by fever and jaundice due to liver dysfunction, has diverse etiologies, including infectious agents, e.g., viruses, bacteria, and parasites, and non-infectious causes [5–7]. This syndrome is prevalent and more complex in resource-limited settings due to a broader range of etiologies and multiple barriers to effective diagnosis [8, 9].

Viral hepatitis is a leading infectious cause of febrile jaundice and poses a global public health challenge, impacting millions worldwide and contributing to significant morbidity and mortality [10–12]. Hepatitis A (HAV), transmitted via the fecal–oral route, is typically prevalent in regions with inadequate sanitation and limited access to clean water, as has been the case in parts of sub-Saharan Africa. However, with progress in urbanization and development across the continent, some areas may not be as highly endemic for HAV as before [13]. Hepatitis B (HBV) and C (HCV) are bloodborne liver infections which can establish chronicity and lead to severe complications, such as cirrhosis and hepatocellular carcinoma, years or decades later [11, 12]. Both viruses remain significant global health challenges. As of 2022, approximately 254 million people worldwide are chronically infected with HBV, with 65% of cases residing in the African and Western Pacific regions [11]. HCV affects an estimated 58 million individuals globally, with 1.5 million new infections occurring annually [14]. The prevalence of HCV is high in sub-Saharan Africa, with a conservative estimated seroprevalence of 2.30% and substantial variation by country and region [15]. Hepatitis E (HEV) is primarily transmitted via the fecal–oral route through contaminated water (genotypes 1 and 2), although zoonotic transmission (genotypes 3 and 4) is increasingly recognized [16]. The global seroprevalence of HEV is estimated at 12.47% by IgG and 1.47% by IgM in the general population [17], with particularly high prevalence in sub-Saharan Africa [16, 18]. Despite its high burden, the epidemiology of viral hepatitis in Africa remains under-researched, and more comprehensive studies are needed to inform effective public health interventions.

In the Democratic Republic of Congo (DRC), yellow fever is the most extensively investigated cause of febrile jaundice. A previous study revealed that from 2003 to 2010 only approximately 5% of suspected cases were confirmed as yellow fever, while viral hepatitis (A–E) accounted for 43.7% [3]. However, no comprehensive risk factor analysis of demographic and geographic factors for viral hepatitis was conducted in that study. Epidemiological data on viral hepatitis in the DRC is focused on HBV and HCV, while the situation for HAV, HDV, and HEV remains largely unknown [19–24]. This study investigates the seroepidemiology of viral hepatitis (HAV, HBV, HCV, and HEV) among febrile jaundice cases initially suspected to be yellow fever in the DRC. By updating the prevalence data and analyzing contributing factors, this study aims to improve public health interventions targeting viral hepatitis.

## Methods

### Study design and participants

This study utilized a retrospective cross-sectional design to perform a secondary analysis of data and serum samples collected through the DRC yellow fever surveillance system. The samples analyzed were obtained from individuals presenting with acute febrile jaundice, initially suspected of having yellow fever but subsequently testing negative for the disease. Only samples stored at − 20 °C with adequate volume for further testing and corresponding sociodemographic data were included.

Since 2003, the DRC has conducted yellow fever surveillance in alignment with WHO guidelines, which mandate a standardized case definition and compulsory reporting by health districts [25, 26]. Under WHO criteria, a suspected yellow fever case is defined as any individual presenting with acute fever and jaundice within 14 days of symptom onset, with no response to an antimalarial drug or a negative malaria test result. Blood samples from suspected cases are collected at the point of care and sent to the Institut National de Recherche Biomédicale (INRB) in Kinshasa, the reference laboratory for yellow fever in the DRC [27].

Between January 2017 and December 2018, 1,592 suspected yellow fever cases were reported to the INRB, encompassing 25 of the 26 provinces and indicating near-national coverage. Samples originated from 224 health districts (43% of all districts). Of the samples received, serological testing using the CDC Atlanta anti-yellow fever virus IgM ELISA protocol [28] confirmed 70 cases (4.5%), while 1522 samples tested negative. After excluding 283 samples due to insufficient volume or missing data, 1239 high-quality samples were retained for this study.

### Laboratory analyses

Serological tests for viral hepatitis were performed by ELISA in 96-well plates, in which HAV infection was defined by the presence of anti-HAV IgM, acute HBV infection by the detection of both hepatitis B surface antigen (HBsAg) and IgM antibodies against the hepatitis B core antigen (anti-HBc IgM), acute HCV infection by the presence of anti-HCV IgM, and HEV by the presence of anti-HEV IgM. Commercial kits from Beijing Wantai Biological Pharmacy Enterprise Co., Ltd., were used according to the manufacturer's instructions. The specific kits used were the Wantai HAV–IgM ELISA, Wantai HBc Ab–IgM ELISA, AiDTM anti-HCV ELISA Plus, Wantai HEV IgM ELISA, and AiDTM HBsAg ELISA. The results were classified based on optical density (OD) ratios. Samples with OD ratios < 0.9 were considered negative, while those with OD ratios > 1.1 were positive. Samples with OD ratios between 0.9 and 1.1 were considered borderline. Borderline results were re-tested in duplicate to confirm the outcome.

### Study variables

The primary outcome variable in this study was the detection of acute viral hepatitis caused specifically by HAV, HBV, HCV or HEV, which was used to estimate their prevalence among people presenting with febrile jaundice in the population. The predictor variables encompassed a range of demographic characteristics including age, sex, and living area; environmental characteristics, such as seasonality and climate type; and geographic characteristics, including province. This comprehensive approach aimed to identify patterns and risk factors associated with acute febrile jaundice due to specific hepatitis viruses, thereby enhancing our understanding of the syndrome and informing public health strategies.

### Statistical analyses

Statistical analyses were performed using the Stata 18 software (StataCorp. 2023. Stata Statistical Software: Release 18. College Station, TX: StataCorp LLC.). Categorical data were summarized as proportions, whereas continuous data were described using the median and interquartile range (IQR). Age was categorized into quartiles. Pearson’s chi-squared test or Fisher’s exact test were used to compare proportions. Univariate and multivariate logistic regression analyses were used to identify and assess the risk factors for viral hepatitis. When analyzing the effect of individual provinces, the odds ratio was calculated using all other provinces as the reference group. The statistical significance threshold was set at p < 0.05 throughout.

## Results

### Sociodemographic and environmental characteristics and hepatitis serology results

A total of 1,239 samples were successfully analyzed in this retrospective study. Table 1 summarizes the sociodemographic and environmental characteristics of the study participants. Among the participants, 729 (58.8%) were male, with a median (IQR) age of 16 [5–31] years, ranging from 0 to 86 years. Most participants, 842 (68%), lived in rural areas, and 906 (73.1%) were identified during the dry season. The most common viral hepatitis was HAV (16.1%), followed by HBV (9.6%), HCV (5.0%) and HEV (3.1%). However, the number of tested cases per 10,000 population varied considerably across provinces from 0 to 11.14 (Appendix Table 1). No cases were tested in Sankuru province, and only one case was tested in each of Maniema and Kasai-Oriental: these provinces were, therefore, excluded from analysis. In contrast, provinces such as Tshuapa and Bas-Uele reported substantially higher numbers of tested cases per population (Appendix Fig. 1). The median (IQR) age also varied from 3 (2–5) years in Ituri (n = 7) to 27.5 (6–47) years in Kasai-Central (n = 18). Participants living in rural areas were significantly younger than those in urban areas: median (IQR) age 12.5 (4–29) vs 20 (8–32) years, respectively (*p* < 0.001).

**Table 1:** Characteristics and prevalence of viral hepatitis A, B, C, and E

Characteristics	Participants (%) (*N* = 1239)
Sex	
Male	729 (58.8)
Female	510 (41.2)
Age, years	16 (5–31)
Age group, years	
0–4	276 (22.3)
5–15	332 (26.8)
16–30	320 (25.8)
> 31	311 (25.1)
Living area	
Rural	842 (67.9)
Urban	397 (32.1)
Seasonal distribution of cases	
Dry season	906 (73.1)
Rainy season	333 (26.9)
Type of climate	
Humid subtropical	208 (16.9)
Tropical rainforest	448 (36.2)
Tropical savanna	582 (46.9)
Years	
2017	757 (61.1)
2018	482 (38.9)
Hepatitis serological results	
Anti-HAV IgM	199 (16.1)
HBs antigen + HBc IgM	119 (9.6)
HBs antigen	424 (34.2)
HBc IgM	139 (11.2)
Anti-HCV IgM	62 (5.0)
Anti-HEV IgM	38 (3.1)

### Characteristics of patients with each hepatitis type

Table 2 summarizes the characteristics of patients according to type of hepatitis virus. There were no associations with gender but several significant associations with age were identified. HAV showed a strong tendency to affect younger children, with a median (IQR) age of 4 (2–7) years: this did not differ between urban and rural areas. In contrast, the youngest age group (0–4 years) was the most protected from HBV. The highest risk age group for HBV was the 16–30 years (aOR 4.60, 95% CI 2.41–8.75), which likely reflects high rates of sexual transmission. The median (IQR) age of HBV patients was 22 (14–32) years. Intriguingly, the risk of HCV was highest in the oldest age group (> = 31 years; aOR 2.95, 95% CI 1.30–6.65). The median (IQR) age of HCV patients was 27 (7–44) years. No association with age was identified for HEV cases, which had a median (IQR) age of 15 (5–29) years.

**Table 2:** Characteristics and risk factors of hepatitis cases by virus type

	HAV IgM positive			HBsAg & HBc IgM positive			HCV IgM positive			HEV IgM positive		
	(n, %)	CrudeOR	Adjusted OR	(n, %)	Crude OR	Adjusted OR	(n, %)	Crude OR	Adjusted OR	(n, %)	Crude OR	Adjusted OR
All study population	199 (16.1)			119 (9.6)			62 (5.0)			38 (3.1)		
Sex												
Female	84 (16.5)	1	1	41 (8)	1	1	26 (5.1)	1	1	19 (3.7)	1	1
Male	115 (15.8)	0.94 (0.69–1.29)	1.11(0.79–1.56)	78 (10.7)	1.37 (0.92–2.03)	1.35 (0.90–2.03)	36 (4.9)	0.96 (0.57–1.62)	0.91(0.53–1.54)	19 (2.6)	0.69 (0.36–1.32)	0.67 (0.34–1.30)
Age group												
0–4	115 (41.7)	12.7 (7.38 21.9)	12.8 (7.37–22.2)	13 (4.7)	1	1	8 (2.9)	1	1	9 (3.3)	1	1
5–15	63 (19.0)	4.17 (2.38 7.31)	4.10 (2.33–7.20)	20 (6.0)	1.29 (0.63 2.65)	1.44(0.70–2.97)	14 (4.2)	1.47 (0.60–3.56)	1.34 (0.55–3.28)	10 (3.0)	0.92 (0.36–2.30)	0.84 (0.33–2.15)
16–30	17 (5.3)	1.00	1	51 (16.0)	3.83 (2.03 7.21)	4.60(2.41–8.75)	13 (4.1)	1.41 (0.57–3.47)	1.24 (0.50–3.07)	10 (3.1)	0.95 (0.38–2.39)	0.95 (0.37–2.46)
> 31	4 (1.3)	0.23 (0.07 0.69)	0.23 (0.07–0.70)	35 (11.3)	2.56(1.32 4.95)	2.86(1.47–5.57)	27 (8.7)	3.18 (1.42–7.13)	2.95 (1.30- 6.65)	9 (2.9)	0.88 (0.34–2.25)	0.82 (0.31–2.16)
Living area												
Urban	42 (10.6)	1	1	26 (6.5)	1	1	27 (6.8)	1	1	8 (2.0)	1	1
Rural	157 (18.6)	1.94 (1.35–2.79)	2.09 (1.31–3.34)	93 (11)	1.77 (1.13–2.78)	1.50 (0.83–2.72)	35 (4.2)	0.59 (0.35–1.00)	0.78 (0.40–1.53)	30 (3.6)	1.80 (0.82–4.00)	1.23(0.45–3.33)
Season of occurrence												
Rainy season	150 (16.6)	1	1	90 (9.9)	1	1	42 (4.6)	1	1	17 (1.9)	1	1
Dry season	49 (14.7)	0.87 (0.61–1.23)	0.93 (0.63–1.37)	29 (8.7)	0.86 (0.56–1.34)	0.75 (0.48–1.19)	20 (6.0)	1.31 (0.76–2.27)	1.37 (0.79–2.40)	21 (6.3)	3.52 (1.83–6.76)	3.28 (1.68–6.37)
Type of climate												
Humid subtropical	36 (17.2)	1.07 (0.69–1.66)	1.21 (0.75–1.97)	25 (11.9)	0.99 (0.60–1.64)	1.06 (0.63–1.78)	8 (3.8)	1.00 (0.43–2.38)	0.92 (0.38–2.21)	16 (7.7)	3.63 (1.62–8.15)	3.40 (1.49–7.74)
Tropical rainforest	73 (16.3)	1	1	54 (12)	1	1	17 (3.8)	1	1	10 (2.2)	1	1
Tropical savanna	90 (15.5)	0.94 (0.67–1.32)	1.86 (1.20–2.9)	40 (6.8)	0.53 (0.35–0.82)	0.59 (0.34–1.02)	37 (6.4)	1.72 (0.96–3.10)	1.46 (0.69–3.05)	12 (2.1)	0.92 (0.39–2.15)	1.07 (0.38–2.97)

Living in a rural area more than doubled the risk of HAV positivity after adjusting for other factors (Table 2; aOR 2.09, 95% CI 1.31–3.34) but no such association was found for the other hepatitis viruses in multivariate analysis.

There was a strong association between HEV cases and the dry season after adjustment for other factors (aOR 3.28, 95% CI 1.68–6.37), whereas none of other viral hepatitides showed distinct seasonality. HEV cases were also associated with a humid subtropical climate and were higher in the year 2018. Notably, there was a discrete cluster of HEV cases from July to August 2018 in Haut-Lomami province (*n* = 5, 38.5%), with four reported in the Songa health zone, and in Lualaba Province (*n* = 7, 31.8%), all of which were reported in the Kapanga health zone. In addition, HAV showed a tendency to occur in a tropical savanna climate (aOR 1.86, 95% CI 1.20–2.9).

### Geographical distribution and risk factors

**Fig. 1:**
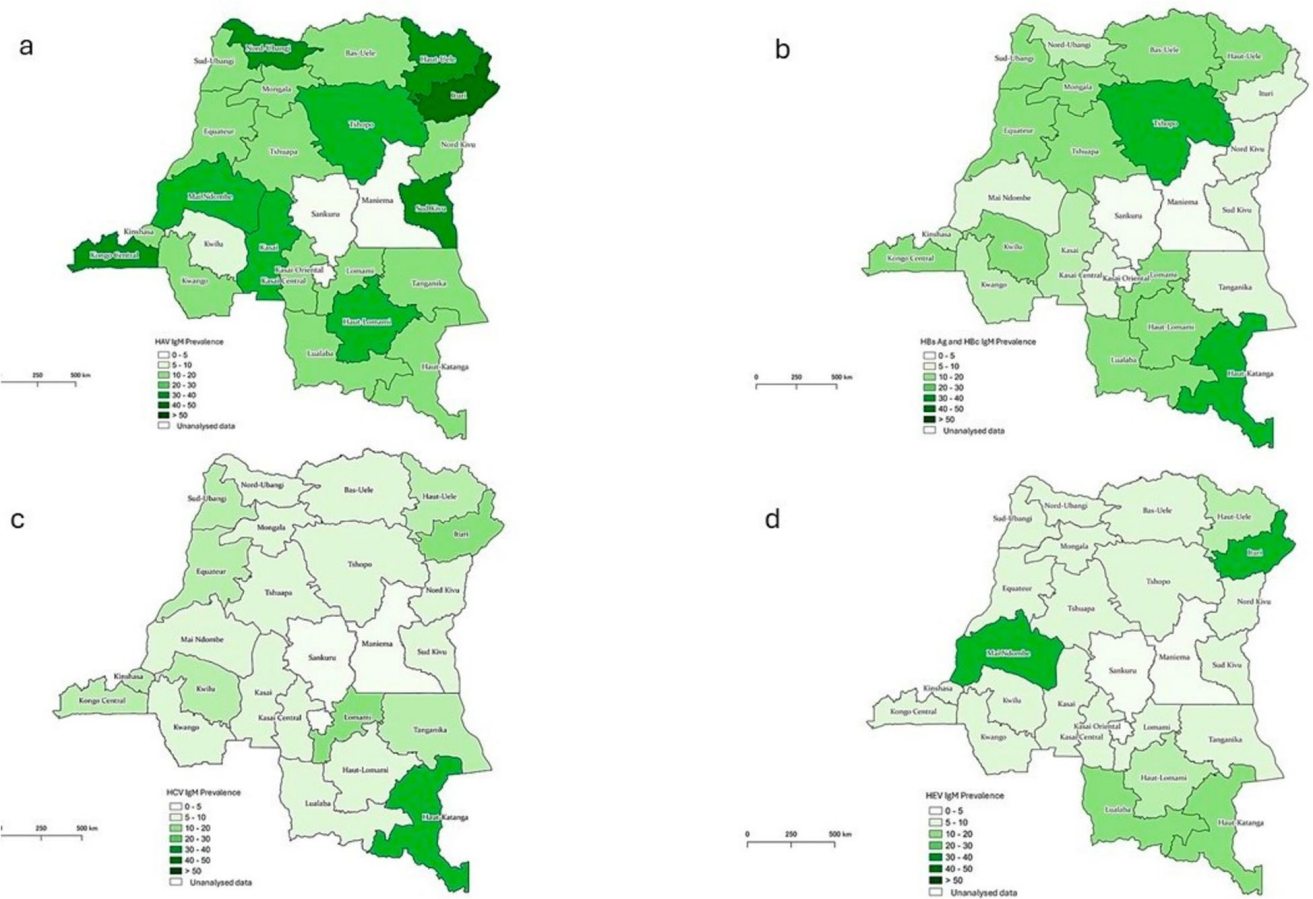
Geographical distribution of viral hepatitis markers in yellow fever suspected patients in the DRC.** a** Geographic distribution of anti-HAV IgM cases. **b** Geographic distribution of HBsAg and anti-HBc IgM-positive cases. **c** Geographic distribution of anti-HCV IgM cases. **d** Geographic distribution of anti-HEV IgM cases

The maps in Fig. 1 illustrate the geographical distribution of the identified viral hepatitis cases in DRC. Detailed data are provided in Appendix Table 1. HAV cases were identified in all the 23 provinces included for analysis. The prevalence of HAV in each province ranged from 3.4 to 39.6% with the median of 17.4% (IQR 14.0–25.0%). Further analysis revealed that HAV cases were more likely detected in at least two provinces, Haut-Uele and Kongo-Central, after adjusting for age group and living area (aOR 7.03, 95% CI 1.40–35.32, *p* = 0.018; aOR 3.19, 95% CI 1.59–6.39, *p* = 0.001, respectively).

HBV cases were identified in 18 (78.3%) of 23 analyzed provinces. The prevalence of HBV in each province ranged from 0% to 28.6% with the median of 11.8% (IQR 3.1–17.2%). HBV positivity was not associated with a particular province. Notably, the five provinces in which HBV was not identified had mostly very young patients, with median ages of 6.5 years or under (Appendix Table 1). Intriguingly, further analysis revealed that febrile jaundice cases from Kinshasa province were significantly less likely to be HBV, after adjusting for age group and living area (aOR 0.36, 95% CI 0.15–0.86, *p* = 0.022).

HCV cases were identified in 17 (73.9%) provinces. The prevalence of HCV in each province ranged from 0% to 28.6% with the median of 4.2% (IQR 3.1–8.7%). HCV cases were more likely to be detected in two provinces, Haut-Katanga and Kongo-Central, after adjusting for age group and living area (aOR 8.56, 95% CI 1.51–48.43, *p* = 0.015; aOR 3.44, 95% CI 1.25–9.44, *p* = 0.016, respectively).

HEV cases were identified in only 13 (56.5%) provinces. The prevalence of HEV in each province ranged from 0% to 28.6% with the median of 1.8% (IQR 0–9.1%). At least three provinces, Haut-Lomami, Ituri, and Lualaba were significantly associated with HEV after adjusting for age group and seasonality (aOR 4.50, 95% CI 1.95–10.39, *p* < 0.001; aOR 14.39, 95%CI 2.45–84.55, *p* = 0.003; aOR 3.95, 95% CI 1.59–9.82, *p* = 0.003, respectively).

## Discussion

This study demonstrated that utilizing residual samples from the national yellow fever surveillance system is a valuable tool not only for determining prevalence but also for gaining comprehensive insights into the demographic and geographical characteristics of viral hepatitis A, B, C, and E.

### Comparison to prevalence data of previous similar studies

Overall, viral hepatitis is a major cause of acute febrile jaundice in the DRC, constituting 35.4% of our tested cases. HAV was the most common, followed by HBV, HCV and HEV. Previous findings from 2003 to 2012 in the same yellow fever surveillance system [3], showed that 43.7% (218/498) of serum samples were infected with at least one of the five investigated hepatitis viruses, including HAV (16.7%), HBV (24.6%), HCV (2.3%), HDV (26.1% of HBV positive patients) and HEV (10.4%). We cannot simply compare these results, since the previous study did not distinguish acute HBV infection from chronic infection. In our study we applied a stringent definition of HBs antigen and anti-HBc IgM positivity to identify a case of acute HBV infection which is likely to be causing the patient’s febrile jaundice. Similar hepatitis studies based on yellow fever surveillance reported variable results: HBsAg (10.6%), HCV IgG/IgM (2.0%), and HEV IgM (16.9%) in Chad, 2019 [29]; HBsAg (19.8%), HCV Ag/IgG/IgM (5.6%), and HEV qPCR (13.6%) among cases randomly selected from surveillance between 2008 and 2010 in the Central African Republic (CAR) [7]. However, not all tests distinguished acute from chronic infection and the diagnostic techniques differed. Furthermore, the sample sizes were relatively small, and the descriptions of yellow fever surveillance were not detailed. There is a need for a standard protocol for hepatitis epidemiology studies based on yellow fever surveillance so that their findings can be rationally compared.

### Complexity of viral hepatitis epidemiology studies in the context of yellow fever surveillance

This study revealed considerable variations in the performance of the national surveillance system across provinces, such as the number of identified cases per population and the age distribution of cases. These variations are likely to reflect true geographical differences in disease distribution, such as HEV. However, it is also plausible that the findings were influenced by socio-economic factors, including conflict and fragile local health systems, as well as the experiences of local health personnel, which may have impacted data collection and reporting for yellow fever surveillance. To ensure reliable statistical analysis, data from three provinces with zero or only one case tested were excluded. Interestingly, Tshuapa and Bas-Uele had the highest number of tests per population. Both provinces had prior experience with Ebola virus disease outbreaks. Such exposure may have enhanced the capacity of local health personnel and strengthened the infectious diseases reporting systems in these regions. Despite these limitations, this study provides several valuable findings to inform and improve public health interventions, although careful interpretation of the results is essential.

### Hepatitis A virus epidemiology in DRC

Patterson et al. [13] estimated a lower anti-HAV IgM prevalence (5.0%) among asymptomatic individuals across Africa. Our study showed that the highest prevalence of HAV by far (41.7%) was among sick children (< 4 years). This underscores the substantial burden of febrile jaundice caused by HAV among young children in Africa and suggests that the true HAV disease burden may be underestimated. Living area significantly influenced HAV prevalence, with rural residents exhibiting a higher prevalence (18.6%) compared to urban residents (10.6%), likely due to fragile infrastructure in rural areas. A study in South Africa similarly found that children are commonly affected by HAV [30]. Further multivariable analysis identified at least two provinces, Haut-Uele and Kongo Central as hotspots. These disparities likely stem from differences in access to clean water, sanitation, and hygiene education, which are particularly inadequate in these regions.

### Hepatitis B virus epidemiology in DRC

The HBV prevalence observed in this study exceeded that in the general population [20, 31], pregnant women [32], and blood donors [21] in DRC. This discrepancy is expected as our study focused on symptomatic individuals. The lowest prevalence was observed in the youngest age group, born nearly a decade after the introduction of the HBV vaccine into the Expanded Programme on Immunization in the year 2007, which reflects the vaccine’s success in reducing HBV infections among children under 5 years in the DRC [31, 33–35]. The increased risk of acute HBV infection among the 16–30 age group suggests that sexual contact is currently the primary mode of HBV transmission in the country. Further analysis revealed that Kinshasa province had a significantly lower HBV prevalence, underscoring disparities in vaccine coverage. These include gaps in administering the birth-dose vaccine, which is crucial for preventing perinatal transmission as well as ongoing horizontal transmission within households [36–39].

### Hepatitis C virus epidemiology in DRC

In the DRC, prevalence of HCV varies widely (0.2–13.7%) [40], and our estimate of 5.0% among people with febrile jaundice exceeds previous reports of 2.3% in DRC [3] and 2.0% in Chad [29]. These differences likely reflect variations in population characteristics and healthcare access, key risk factors for HCV transmission in Africa. We found that age was associated with HCV positivity, with the oldest age group > = 31 years showing the highest risk. It is likely due to unsafe medical and other practices, including blood transfusions with unscreened blood, and traditional procedures including scarification with unsterilized equipment [41–43]. Geographic disparities in HCV prevalence were notable, with the provinces of Haut Katanga (28.6%) and Kongo Central (10.4%) showing significantly higher rates in adjusted analysis.

### Hepatitis E virus epidemiology in DRC

Our estimated 3.1% HEV prevalence among febrile jaundice patients is consistent with the 2.6% prevalence reported in Burkina Faso [44] but lower than that reported in other African studies. For instance, among patients with suspected yellow fever, anti-HEV IgM seroprevalence was 8.1% in Cameroon [45] and 22.6% in the CAR [46]. These differences are expected as HEV prevalence is likely affected by variations in seasonality, years, study populations, sociocultural factors, and the predominance of endemic vs epidemic transmission patterns. Our results demonstrated significant seasonal variation, with increased seropositivity during the dry season (6.3 vs 1.9%) and yearly variation (0.4% in 2017 and 7.3% 2018). We attributed this to reduced water availability and deteriorating sanitary conditions, leading to a higher concentration of HEV in water sources. However, this finding contrasts with previous studies reporting increased risk during rainy seasons due to fecal contaminants in water sources during heavy rainfall and increased exposure to animal reservoirs of HEV, particularly pigs [46–48]. The interplay of seasonal variation and HEV transmission is made more complex by genotype variations. Further multivariable analysis identified three provinces with a higher risk of HEV: Haut-Lomami (9.6%, 8 out of 83 cases), Ituri (28.6%, 2 out of 7 cases), and Lualaba (12.3%, 7 out of 57 cases). The observed clustering of HEV cases in two specific health zones, notably Kapanga and Songa within these provinces, indicates local disparities in factors including water, sanitation, and hygiene. Geographic heterogeneity further emphasizes the importance of region-specific strategies to mitigate HEV burdens and conducting molecular epidemiological investigations.

## Limitations

First, viral hepatitis epidemiological data in this study is biased by yellow fever surveillance, which is facility-based and, therefore, does not reflect community-level virus dynamics. However, since yellow fever surveillance has been conducted across multiple yellow fever endemic countries over several years, our findings suggest that leveraging this surveillance should enhance our understanding of viral hepatitis epidemiology. Second, this study did not investigate hepatitis D. However, as HDV infection does not cause jaundice in the absence of HBV co-infection, it is unlikely to have contributed to underestimation of the total prevalence of viral hepatitis. Third, we did not assess the impact of co-infection as the number of co-infection cases was too small for analysis. Fourth, this study did not explore other causes of febrile jaundice. Although we previously investigated leptospirosis [27], testing a large number of samples for novel pathogens remains costly. The development of multi-valent serological tests is warranted.

## Conclusion

This study demonstrates that viral hepatitis is a major cause of acute febrile jaundice in the DRC, with notable geographic and seasonal trends. Tailored interventions are essential to mitigate the burden of viral hepatitis in each province. The national yellow fever surveillance serves as a valuable resource for understanding viral hepatitis epidemiology and holds potential for improving public health interventions at the provincial level. However, its considerable variations in performance necessitate careful interpretation. Establishing international guidelines for conducting viral hepatitis epidemiological analysis using yellow fever surveillance data is crucial.

## Supplementary Information

Supplementary Material 1

Supplementary Material 2

## Data Availability

Data is provided within the manuscript or supplementary information files.

## Abbreviations

aOR: Adjusted odds ratio
CAR: Central African Republic
CDC: Centers for disease control and prevention
CI: Confidence interval
DRC: Democratic Republic of the Congo
ELISA: Enzyme-linked immunosorbent assay
HAV: Hepatitis A virus
HBcAg: Hepatitis B core antigen
HBc IgM: Hepatitis B core IgM antibody
HBsAg: Hepatitis B surface antigen
HBV: Hepatitis B virus
HCV: Hepatitis C virus
HDV: Hepatitis D virus
HEV: Hepatitis E virus
IgG: Immunoglobulin G
IgM: Immunoglobulin M
INRB: Institut National de Recherche Biomédicale
IQR: Interquartile range
OD: Optical density
qPCR: Quantitative polymerase chain reaction
