# Supplementary figures and images for: Revealing viral hepatitis epidemiology in the Democratic Republic of Congo: insights from yellow fever surveillance reanalysis
Source: Trop Med Health. 2025 Feb 5;53:17. doi: 10.1186/s41182-025-00687-8 (PMC11800490; doi:10.1186/s41182-025-00687-8)

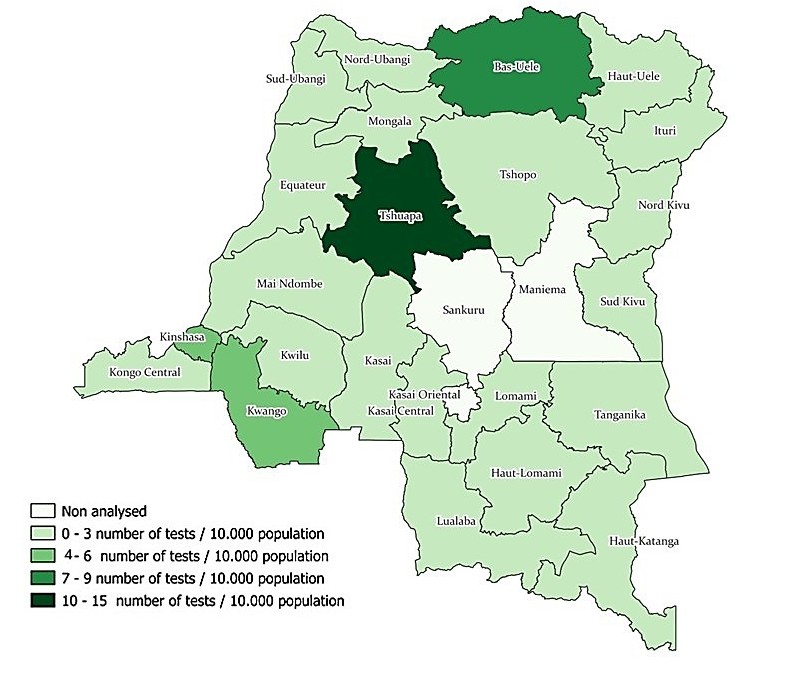

Supplement: Supplementary file 1 — Supplementary Material 1 [file 41182_2025_687_MOESM1_ESM.jpg]
