# Supplementary material for: Revealing viral hepatitis epidemiology in the Democratic Republic of Congo: insights from yellow fever surveillance reanalysis
Source: Trop Med Health. 2025 Feb 5;53:17. doi: 10.1186/s41182-025-00687-8 (PMC11800490; doi:10.1186/s41182-025-00687-8)
Supplement: Supplementary file 2 — Supplementary Material 2 [file 41182_2025_687_MOESM2_ESM.docx]

**Appendix Table 1. Characteristics and Risk Factors of Hepatitis Cases by Virus Type**

| Province | Population  in 2018 | Urban% | number of tested cases | n of tests/10,000 population | Median age (years) | 25%ile | 75%ile | anti-HAV IgM | HAV% | HBc IgM  & HBsAg | HBV% | HCV IgM | HCV% | anti-HEV IgM | HEV% |
| --- | --- | --- | --- | --- | --- | --- | --- | --- | --- | --- | --- | --- | --- | --- | --- |
| Tshuapa | 2002087 | 0.0 | 223 | 11.14 | 21 | 6 | 38 | 25 | 11.2 | 24 | 10.8 | 6 | 2.7 | 4 | 1.8 |
| Bas-Uele | 1205278 | 0.0 | 92 | 7.63 | 18.5 | 4 | 28 | 17 | 18.5 | 15 | 16.3 | 4 | 4.3 | 3 | 3.3 |
| Kinshasa | 9053734 | 97.7 | 342 | 3.78 | 20 | 9 | 32 | 36 | 10.5 | 19 | 5.6 | 24 | 7.0 | 6 | 1.8 |
| Kwango | 2363401 | 16.7 | 84 | 3.55 | 11.5 | 5 | 25 | 15 | 17.9 | 6 | 7.1 | 3 | 3.6 | 0 | 0.0 |
| Nord-Ubangi | 1494249 | 0.0 | 48 | 3.21 | 4.5 | 2 | 13.5 | 18 | 37.5 | 3 | 6.3 | 2 | 4.2 | 2 | 4.2 |
| Lualaba | 2171930 | 0.0 | 57 | 2.62 | 25 | 9 | 37 | 8 | 14.0 | 8 | 14.0 | 1 | 1.8 | 7 | 12.3 |
| Equateur | 2313031 | 11.8 | 51 | 2.20 | 8 | 3 | 17 | 7 | 13.7 | 6 | 11.8 | 3 | 5.9 | 1 | 2.0 |
| Haut-Lomami | 3774201 | 21.7 | 83 | 2.20 | 10 | 4 | 27 | 18 | 21.7 | 10 | 12.0 | 0 | 0.0 | 8 | 9.6 |
| Kongo-Central | 3759176 | 0.0 | 48 | 1.28 | 8 | 4 | 21.5 | 19 | 39.6 | 6 | 12.5 | 5 | 10.4 | 0 | 0.0 |
| Sud-Ubangi | 2712829 | 17.4 | 23 | 0.85 | 11 | 2 | 36 | 4 | 17.4 | 4 | 17.4 | 2 | 8.7 | 0 | 0.0 |
| Haut-Uele | 1742337 | 0.0 | 11 | 0.63 | 20 | 6 | 48 | 4 | 36.4 | 2 | 18.2 | 1 | 9.1 | 1 | 9.1 |
| Kwilu | 4730112 | 27.6 | 29 | 0.61 | 20 | 9 | 29 | 1 | 3.4 | 5 | 17.2 | 2 | 6.9 | 1 | 3.4 |
| Lomami | 3726570 | 9.1 | 22 | 0.59 | 24 | 6 | 38 | 3 | 13.6 | 4 | 18.2 | 3 | 13.6 | 0 | 0.0 |
| Kasaï-Central | 4504811 | 0.0 | 26 | 0.58 | 6 | 3 | 15 | 4 | 15.4 | 0 | 0.0 | 1 | 3.8 | 0 | 0.0 |
| Tanganyika | 2925675 | 0.0 | 13 | 0.44 | 6 | 4 | 11 | 2 | 15.4 | 0 | 0.0 | 1 | 7.7 | 0 | 0.0 |
| Kasaï | 4226832 | 0.0 | 18 | 0.43 | 27.5 | 6 | 47 | 4 | 22.2 | 1 | 5.6 | 0 | 0.0 | 0 | 0.0 |
| Nord-Kivu | 9596812 | 21.9 | 32 | 0.33 | 17 | 6.5 | 20.5 | 5 | 15.6 | 1 | 3.1 | 1 | 3.1 | 1 | 3.1 |
| Mongala | 2381882 | 0.0 | 7 | 0.29 | 4 | 3 | 6 | 1 | 14.3 | 1 | 14.3 | 0 | 0.0 | 0 | 0.0 |
| Maï-Ndombe | 1815544 | 0.0 | 4 | 0.22 | 5.5 | 3.5 | 13.5 | 1 | 25.0 | 0 | 0.0 | 0 | 0.0 | 1 | 25.0 |
| Tshopo | 3012809 | 0.0 | 4 | 0.13 | 12.5 | 6 | 21 | 1 | 25.0 | 1 | 25.0 | 0 | 0.0 | 0 | 0.0 |
| Ituri | 5351951 | 0.0 | 7 | 0.13 | 3 | 2 | 5 | 3 | 42.9 | 0 | 0.0 | 1 | 14.3 | 2 | 28.6 |
| Haut-Katanga | 5653009 | 28.6 | 7 | 0.12 | 7 | 2 | 32 | 1 | 14.3 | 2 | 28.6 | 2 | 28.6 | 1 | 14.3 |
| Sud-Kivu | 6658251 | 33.3 | 6 | 0.09 | 6.5 | 3 | 10 | 2 | 33.3 | 0 | 0.0 | 0 | 0.0 | 0 | 0.0 |
| Maniema | 2470780 | 0 | 1 | 0.04 | NA | NA | NA | NA | NA | NA | NA | NA | NA | NA | NA |
| Kasaï-Oriental | 5053395 | 0 | 1 | 0.02 | NA | NA | NA | NA | NA | NA | NA | NA | NA | NA | NA |
| Sankuru | 1911390 | NA | 0 | 0.00 | NA | NA | NA | NA | NA | NA | NA | NA | NA | NA | NA |
